# A study of push and pull factors influencing employee retention in education sector in China: Using PLS-SEM and multi-group analysis

**DOI:** 10.1371/journal.pone.0349605

**Published:** 2026-05-18

**Authors:** Zhengda Yao, Juanjuan Zhang, Dejun Kang

**Affiliations:** 1 Department of Human Resources, Guang’an Institute of Technology, Guang’an City, Sichuan Province, China; 2 Graduate School of Business, SEGi University of Malaysia, Petaling Jaya, Selangor Darul Ehsan, Malaysia; Akademia Jagiellońska w Toruniu: Akademia Jagiellonska, CZECHIA

## Abstract

Employee retention remains a pressing issue and it reflects a key psychological decision-making outcome shaped by work-related stressors and organizational resources. Drawing on the Push-Pull framework, this study examines how pull factors (training and development, compensation, and empowerment) and push factors (job dissatisfaction, job burnout, and peer turnover influence) affect employee retention, while testing the moderating role of engagement. Using survey data from 568 educators in Guangdong Province, Partial Least Squares Structural Equation Modeling and Multi-Group Analysis were employed. Results show that pull factors positively predict employee retention, whereas push factors negatively affect it. Engagement moderates the relationship between training and development and employee retention. The multi-group analysis results indicate that only job burnout shows a significant difference between public and private schools. The findings are important for education policymakers and school administrators. Targeted retention policies that integrate material incentives and psychological engagement can help stabilize the teaching workforce.

## Introduction

Employee retention has become a central issue in the education sector worldwide, as high turnover not only undermines institutional stability but also negatively impacts student outcomes and educational quality [1,2]. Recent labor market data from China show that the retention rate in the education industry declined to 79.6% in 2024, which is lower than that of many other major industries and highlights the urgency of addressing retention issues among educators [3]. These trends are consistent with international evidence suggesting that the education sector experiences higher turnover compared to comparable professions due to unique job demands and emotional labor requirements [1].

In Guangdong Province, retention challenges are compounded by rapid labor mobility and competitive job markets. Guangdong’s education system serves a large and diverse workforce encompassing public, private, and international schools, creating both opportunities and pressures for educators [4]. Demographic changes-such as uneven population growth across regions-have contributed to staffing fluctuations, with some districts experiencing teacher surpluses while others face shortages [4]. Furthermore, performance evaluation reforms and rising expectations for professional competencies have intensified workload stress among teaching staff, which has been widely associated with reduced employee retention [5].

Research indicates that job dissatisfaction, emotional exhaustion, and lack of professional support significantly reduce employee retention in education [6]. In particular, burnout has been identified as a strong predictor of teachers’ attrition intentions, reflecting sustained psychological strain and diminished work engagement [6,7]. Additionally, inadequate organizational support and limited career development opportunities further weaken institutional commitment and increase attrition risk [4,7]. These psychosocial and environmental factors together create a climate in which many educators feel less inclined or able to remain in their positions.

Despite robust research on individual predictors of retention, studies rarely integrate both negative (push) and positive (pull) factors within a unified theoretical framework [8]. The push-pull framework, which originally applied in migration and mobility research, offers a comprehensive perspective by conceptualizing retention outcomes as the result of opposing forces that either push employees away (burnout; job dissatisfaction; peer turnover influence) or pull them toward staying (e.g., training and development, fair compensation; empowerment) [9]. Although this framework has been applied in broader research, its application in the education sector—particularly in rapidly changing regional contexts such as Guangdong—remains underdeveloped. Moreover, the literature remains divided regarding the relative importance of psychological versus organizational determinants of employee retention, with some studies emphasizing structural factors such as compensation and working conditions [10], while others highlight psychological states such as engagement and burnout as more influential drivers [11]. This inconsistency suggests a lack of consensus in explaining retention mechanisms. Additionally, prior research often assumes homogeneous effects across populations, overlooking potential heterogeneity in retention mechanisms across subgroups (e.g., school type or gender) [12].

To address these gaps, the present study applies a push-pull theoretical framework to explore determinants of employee retention in the education sector in Guangdong Province. We adopt a hybrid approach using Partial Least Squares Structural Equation Modeling (PLS-SEM) to estimate complex paths among multiple push and pull factors simultaneously [13,2]. In addition, Multi-Group Analysis (MGA) is used to identify whether these relationships differ across key subgroups, such as school type or gender, thereby uncovering heterogeneity in retention mechanisms [13]. This methodological combination not only enhances the robustness of findings but also yields practical insights for tailored retention strategies.

In summary, this study aims to extend the literature on employee retention by integrating both push and pull influences within a single analytical model and examining subgroup differences in a high-mobility educational labor market context. The study’s objectives are achieved through empirical testing of the proposed framework and subgroup comparisons, providing both theoretical contributions and actionable recommendations for educational administrators and policymakers seeking to improve retention outcomes in the face of mounting workforce challenges.

### Employee retention

Employee retention is defined as an organization’s ability to maintain a sustained employment relationship between teachers and schools [14]. Employee retention contributes to effective human resource planning by enabling organizations to anticipate gaps between future workforce demand and supply in line with institutional goals [6]. Within education system, teacher retention is not only essential for maintaining workforce stability but also directly affects teaching quality, student learning outcomes, and the realization of educational equity objectives [15]. As educational reforms continue to deepen, heightened performance evaluation standards and the accumulation of teaching and administrative responsibilities have substantially increased teachers’ occupational stress, making teacher retention a pressing issue in educational management research [6].

Existing studies indicate that employee retention in the education sector carries not only significant economic implications but also important social and institutional value. Teacher attrition is often accompanied by rising hidden costs, including disruptions to instructional continuity, increased training expenses for new teachers, and the loss of organizational knowledge [16]. In the Chinese context, replacement costs are particularly pronounced, especially in compulsory education and private schooling sectors, where frequent personnel mobility may further exacerbate regional inequalities in the allocation of educational resources [4].

From a managerial perspective, teacher retention is not the outcome of a single policy but rather the result of the combined effects of multiple management practices, including compensation incentives, work environment, career development opportunities, and organizational support [17]. Research suggests that teachers’ retention intentions are significantly strengthened when they are granted greater autonomy in instructional decision-making, receive continuous professional development support, and perceive performance evaluation and reward systems as fair [4]. Conversely, burnout, emotional exhaustion, peer turnover spillover effects, and limited promotion opportunities tend to weaken organizational commitment and intensify teachers’ intentions to leave [6]. Consequently, scholars widely argue that organizations must expand and implement diversified teacher retention strategies to enhance organizational resilience and effectively address employee turnover and attrition [14]. Teacher retention should not rely solely on post-entry or tenure-stage management practices but should be embedded throughout the entire career development cycle, encompassing professional support, performance feedback, and long-term incentive mechanisms [14].

Against the backdrop of fiscal constraints and structural reforms within China’s education system, the continuous entry of younger teachers into the profession has intensified schools’ need to retain high-quality teaching staff. Studies indicate that strengthening teachers’ participation in instructional decision-making and school governance can enhance organizational identification and retention intentions [18]. Accordingly, both educational authorities and schools must adopt systematic and effective management measures to mitigate teacher attrition risks and maintain workforce stability [18]. Prior research further suggests that the costs associated with teacher turnover extend beyond direct recruitment and training expenses, with total replacement costs often reaching up to 2.5 times an individual teacher’s salary, potentially generating long-term negative consequences for instructional continuity and school reputation [19].

Based on the above considerations, there is a clear need for systematic research within the Chinese education context to examine the effects of key factors such as work-life balance, work environment, and reward and compensation systems on teacher retention. Such research can provide empirical evidence to support the development of more scientifically grounded and context-sensitive teacher retention strategies for educational organizations.

### Push-pull framework

The push-pull framework, first formalized by Everett Lee in 1966 for human migration, identifies “push” factors (negative conditions repelling individuals from a location or role) and “pull” factors (positive attractions drawing them elsewhere) [20]. It has since adapted to explain labor market behaviors, including teacher mobility, where school working conditions (e.g., administrative support, discipline enforcement) act as malleable pushes or pulls influencing retention or transfer decisions [21].

One of the key characteristics of the Push-Pull framework is its flexibility, which allows researchers to freely define push and pull variables according to different research contexts. For example, push and pull factors may include job stress, organizational support, career development opportunities, compensation, and work-life balance. This contextual adaptability of variable selection enables the Push-Pull framework to provide theoretical explanations and empirical testing across industries and behavioral domains.

In the present study, the classification of variables into push and pull factors is based on their underlying psychological and behavioral functions in the retention process. Push factors are defined as negative work-related conditions that increase dissatisfaction, emotional exhaustion, or social pressure, thereby driving employees away from the organization. Accordingly, variables such as job dissatisfaction, burnout, and peer turnover intention are conceptualized as push factors, as they reflect adverse experiences that weaken employees’ attachment to the organization.

In contrast, pull factors are defined as positive organizational resources that enhance the perceived attractiveness of staying and strengthen employees’ motivation to remain. Therefore, variables including training and development, compensation, and empowerment are categorized as pull factors, as they provide career opportunities, resource support, and intrinsic motivation that encourage long-term retention.

Consistent with this classification logic, prior studies in organizational behavior and employee retention have identified burnout, pay imbalance, and limited career development as push factors, while career advancement opportunities, training support, and leadership support function as pull factors [22,23]. The Push-Pull framework has also been applied in research on nurse retention [24], talent development [25], and user switching behavior [26], demonstrating its ability to explain both the drivers of leaving and the mechanisms of staying.

In the context of the education sector, the Push-Pull framework provides a useful lens for understanding teacher retention [9]. Push factors typically manifest as negative work experiences, such as heavy workloads, burnout, declining job satisfaction, and social comparison pressures from colleagues leaving [21], whereas pull factors include training and development opportunities, fair compensation, supportive leadership, and favorable work-life balance policies [22,12].

Despite these advances, existing studies have often examined antecedents of teacher turnover or retention in isolation. Research that integrates both push and pull factors within a unified analytical framework remains limited, particularly in the context of regionalized education in China [8]. Moreover, prior literature tends to assume homogeneity among teachers and pays insufficient attention to differences across school types, gender, or career stages [12]. Therefore, this study systematically integrates key push and pull factors within a unified framework to more comprehensively reveal the mechanisms underlying teacher retention in the Chinese educational context.

#### Influence of pull factor on employee retention.

Within the push-pull framework, “pull factors” are typically defined as attractive characteristics or resources within an organization, such as compensation and benefits, development opportunities, and organizational support. These positive attributes enhance job appeal, guiding individuals to choose to “stay,” thereby improving employee retention rates [27,28].

#### Training and development.

Training and development refer to organizational practices that enhance employees’ professional skills, pedagogical knowledge, and career development opportunities [29]. Within the Push-Pull framework, training and development function as a key pull factor by increasing the perceived benefits of staying in the organization and strengthening employees’ long-term career expectations [30].

Prior studies consistently show that comprehensive induction (mentoring, coaching, and targeted PD) significantly lowers the probability that new teachers leave their schools or the profession, consistent with the idea that high-quality development increases the perceived returns to staying [31]. Such opportunities signal organizational investment in teachers’ professional growth, which fosters a reciprocal sense of loyalty in line with social exchange theory [32].

Empirical evidence further suggests that insufficient training and limited career advancement opportunities are among the primary reasons driving teachers’ turnover, especially in contexts undergoing rapid educational reform [33]. In contrast, schools that provide structured professional development, continuous learning opportunities, and clear promotion mechanisms tend to report higher retention levels, even under conditions of high job demands [34].

In the Chinese education context, training and development opportunities are closely integrated with teacher evaluation and title promotion systems as well as policy-driven capacity-building initiatives; when teachers perceive these pathways as accessible and effective, they tend to report higher organizational commitment and a stronger intention to remain in their current positions [35]. Therefore, training and development serve as a critical pull factor enhances employee retention in the education sector.

Based on the theoretical reasoning and empirical evidence, the following hypothesis is proposed:

**H1: Training and development positively influence employee retention in the education sector.**

#### Compensation.

Compensation refers to the total monetary and non-monetary rewards that employees receive in return for their work, including salary, bonuses, allowances, and welfare benefits [36]. In the Push-Pull framework, compensation constitutes a key pull factor that enhances the perceived economic returns to staying in the organization. More favorable pay and benefits shift teachers’ benefit evaluations toward retention, thereby increasing the likelihood that they choose to remain rather than exit [37].

Prior research consistently indicates that fair and competitive compensation is positively associated with employee retention, as it reflects organizational recognition, distributive justice, and long-term employment value [38]. In the education sector, compensation plays a particularly salient role because teaching positions often involve high workloads and emotional demands but relatively constrained income growth, making pay-related perceptions especially influential in retention decisions [10].

Empirical evidence further suggests that inadequate compensation or perceived pay inequity significantly increases teachers’ turnover intentions, whereas transparent and performance-aligned compensation systems contribute to stronger organizational commitment and retention [15]. From a social exchange perspective, equitable compensation signals organizational support and respect, thereby encouraging reciprocal commitment from teachers [32].

In the Chinese education context, disparities in salary structures across school types and regions further amplify the role of compensation as a key retention mechanism. Studies indicate that inadequate or unfair compensation can weaken teachers perceived organizational support and accelerate attrition, whereas transparent and performance-linked compensation systems enhance retention intentions [15].

Based on the theoretical reasoning and empirical evidence, the following hypothesis is proposed:

**H2: Compensation positively influences employee retention in the education sector.**

##### Empowerment:

Empowerment refers to the extent to which employees are granted autonomy, authority, and meaningful participation in work-related decision-making processes, enabling them to exercise control over their tasks and professional practices [39]. Within the Push-Pull framework, empowerment functions as a crucial pull factor by enhancing employees’ perceived control, professional value, and psychological attachment to the organization, thereby increasing the attractiveness of remaining in their current positions [40].

Research demonstrates that empowered employees tend to exhibit higher levels of job satisfaction, organizational commitment, and work engagement, all of which are key antecedents of employee retention [41]. In the education sector, teacher empowerment-manifested through instructional autonomy, participation in school governance, and influence over curriculum and assessment decisions-has been shown to significantly reduce turnover intentions and strengthen teachers’ intention to stay [42].

Empirical evidence further suggests that limited decision-making authority and excessive administrative control undermine teachers’ sense of professional identity, thereby increasing emotional exhaustion and attrition risk [43]. In contrast, schools that promote participatory leadership and shared decision-making structures create a supportive organizational climate that enhances teachers perceived organizational support and long-term commitment [44].

Studies indicate that when teachers perceive higher levels of instructional autonomy and involvement in school affairs, they report stronger organizational identification and a higher intention to remain, even amid heightened performance evaluation pressures [45]. Therefore, empowerment serves as a critical pull factor that enhances employee retention in the education sector.

Based on the theoretical reasoning and empirical evidence, the following hypothesis is proposed:

**H3: Empowerment positively influences employee retention in the education sector.**

#### Influence of push factors on employee retention.

Push factors refer to negative workplace conditions or experiences that decrease employees’ willingness to remain in their current organization. In the context of teacher retention, these factors commonly include heavy workload, job dissatisfaction, burnout, and social comparison processes [46,8,47,21,12].

Within the Push-Pull framework, such factors operate by weakening teachers’ organizational commitment and reducing the perceived attractiveness of staying, thereby increasing the likelihood of turnover. In this research, job dissatisfaction reflects a cognitive evaluation of unfavorable work conditions, burnout captures a state of psychological and emotional exhaustion, and peer turnover influence represents a socially driven process shaped by colleagues’ behaviors. Together, these constructs constitute complementary push mechanisms operating at cognitive, psychological, and social levels.

#### Job dissatisfaction.

Job dissatisfaction refers to an employee’s negative cognitive evaluation of their work experience, often accompanied by unfavorable emotional responses [48]. In the education sector, teachers who experience low job satisfaction are more likely to feel disengaged from their schools, experience reduced motivation, and consider leaving their positions [6,7]. Empirical studies indicate that job dissatisfaction is one of the strongest predictors of teacher turnover intentions, as it reflects a perceived misalignment between teachers’ expectations and their actual work experiences [49].

Unlike job burnout, which reflects an affective state of psychological exhaustion, job dissatisfaction is primarily evaluative in nature and rooted in individuals’ cognitive judgments about their work conditions. Teachers may feel dissatisfied due to unmet expectations regarding recognition, fairness, or career development, even in the absence of severe emotional depletion.

Within the Push-Pull framework, job dissatisfaction functions as a cognitive push factor by shaping employees’ overall evaluation of whether their current job meets their expectations. When teachers perceive misalignment in areas such as recognition, evaluation, or career support, they are more likely to reassess their decision to remain in the organization. In this sense, job dissatisfaction “pushes” teachers away by undermining organizational commitment and reducing the perceived value of staying, rather than through psychological strain or social influence [8,21].

Therefore, job dissatisfaction operates primarily through a cognitive evaluative process, distinguishing it from burnout, which operates through affective depletion.

Based on the theoretical reasoning and empirical evidence, the following hypothesis is proposed:

**H4: Job dissatisfaction negatively influences employee retention in the education sector.**

#### Job burnout.

Job burnout refers to a psychological state of emotional exhaustion, depersonalization, and reduced personal accomplishment resulting from prolonged exposure to work-related stress [50]. In the education sector, teachers frequently encounter sustained job demands such as heavy workloads, time pressure, and administrative responsibilities, which gradually deplete their emotional and cognitive resources [6,7].

Unlike job dissatisfaction, which reflects a cognitive evaluation of one’s job, burnout captures an affective state of psychological depletion. Teachers experiencing burnout are not merely dissatisfied but emotionally exhausted and less capable of engaging effectively in their work. Empirical evidence consistently shows that burnout is a strong predictor of turnover intentions, as emotionally drained teachers are more likely to withdraw from their roles and consider leaving the profession [51].

Within the Push-Pull framework, burnout operates as an affective push factor rooted in psychological strain. It “pushes” teachers away by reducing intrinsic motivation and work engagement rather than by altering job evaluations. Moreover, burnout may intensify the negative effects of other factors, such as job dissatisfaction, by lowering individuals’ coping capacity [52].

Therefore, burnout operates primarily through an affective depletion mechanism, distinguishing it from job dissatisfaction, which is based on cognitive evaluation.

Based on the theoretical reasoning and empirical evidence, the following hypothesis is proposed:

**H5: Job burnout negatively influences employee retention in the education sector.**

#### Peer turnover influence.

Peer turnover influence refers to the social and contextual impact of colleagues’ departure on an individual teacher’s retention decision. Unlike job dissatisfaction and burnout, which originate from personal evaluations or psychological states, peer turnover influence is externally driven and embedded in the social environment. It reflects how individuals interpret and respond to the behaviors of others in the workplace [47].

Drawing on social influence and social learning theories, employees tend to model the behaviors and decisions of their peers, especially under conditions of uncertainty [53]. The departure of colleagues may serve as a social signal indicating underlying organizational problems or better external opportunities, thereby shaping individual attitudes toward staying or leaving.

Within the Push-Pull framework, peer turnover influence functions as a socially transmitted push factor. Rather than directly increasing stress or dissatisfaction, it indirectly “pushes” teachers by altering their perceptions of organizational stability, career prospects, and normative behavior. In school settings, high levels of peer turnover can weaken collegial ties, reduce morale, and trigger social comparison processes, all of which may contribute to increased turnover intentions [47]. This effect is particularly salient in regionalized education systems, where mobility patterns are more visible and influential [4].

Based on the theoretical reasoning and empirical evidence, the following hypothesis is proposed:

**H6: Peer turnover influence negatively influences employee retention in the education sector.**

#### Moderating role of engagement.

Engagement refers to a positive, fulfilling, and work-related psychological state characterized by vigor, dedication, and absorption [54].

From a theoretical perspective, engagement plays a crucial moderating role in the relationship between organizational practices and employee retention. According to the Job Demands-Resources (JD-R) model, employees with higher engagement are more capable of converting organizational resources—such as training and development opportunities—into positive work attitudes and long-term commitment [11]. In contrast, low engagement may weaken the effectiveness of such practices, as employees are less likely to internalize or fully utilize development opportunities provided by the organization.

Empirical studies support this interaction mechanism by showing that training and development initiatives are more strongly associated with retention outcomes among highly engaged employees, who tend to perceive professional development as an investment in their long-term career [55]. In the education sector, engaged teachers are more likely to actively participate in professional development activities, apply newly acquired skills in teaching practice, and develop stronger organizational attachment, thereby amplifying the retention-enhancing effects of training and development [51].

In the Chinese education context, ongoing curriculum reforms and performance-based evaluation systems have increased the importance of teacher engagement as a psychological buffer [56]. When teachers exhibit high engagement, training and development opportunities are more likely to be interpreted as supportive and empowering, strengthening their intention to remain. Conversely, under low engagement, similar initiatives may fail to produce meaningful retention effects [11]. Therefore, engagement is expected to condition the strength of the relationship between training and development and employee retention.

Based on the theoretical reasoning and empirical evidence, the following hypothesis is proposed:

**H7: Engagement moderates the relationship between training and development and employee retention in the education sector.**

**Fig 1:**
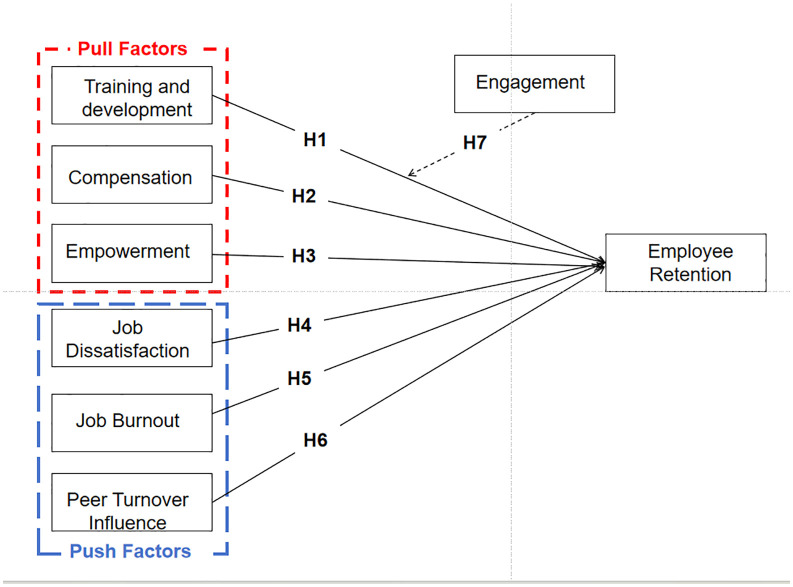
Hypothesis Framework.

Hypothesis framework presents in Fig 1.

## Methodology

### Research design

This study adopted a quantitative, cross-sectional research design to examine employee retention in the education sector. Data were collected using a questionnaire survey and a purposive sampling approach, which enabled the targeted selection of education professionals with relevant work experience and organizational exposure [57].

All measurement items were adapted from previously validated scales in the employee retention and organizational behavior literature. Training and development (4 items), compensation (4 items), empowerment (5 items), employee engagement (4 items), and employee retention (4 items) were measured using scales adapted from Chatzoudes and Chatzoglou [58]. Job dissatisfaction was measured using four negatively worded items adapted from established job satisfaction measures following the operationalization approach of Chatzoudes and Chatzoglou [58]. Burnout was measured using four items adapted from Yuliana et al. [59], and peer turnover influence was measured using three items adapted from Fu et al. [60].

To ensure content validity and contextual suitability for the education sector, the questionnaire was reviewed by academic experts and subsequently pilot-tested among education employees with similar demographic characteristics to the target sample. Minor revisions were made based on the feedback received to improve clarity and reliability.

The final questionnaire consisted of two sections: demographic information and construct measurements related to push and pull factors as well as employee retention. All items were measured using a seven-point Likert scale ranging from 1 (strongly disagree) to 7 (strongly agree), as this format provides greater sensitivity and reliability in capturing employees’ attitudinal and behavioral responses [61]. The full questionnaire instrument and measurement items are provided in the Supporting Information to facilitate reproducibility and enable independent validation of the study.

### Sampling and sample size determination

A purposive sampling strategy was employed. Following the minimum sample size estimation guidelines for PLS-SEM proposed by Kock and Hadaya (2018), both the inverse square root and gamma-exponential methods were applied [62]. With seven predictors in the structural model and a minimum detectable path coefficient of 0.15, the required sample size was estimated at approximately 197 and 209 cases, respectively. Consistent with SEM methodological recommendations suggesting a minimum of 200–300 observations for models with multiple latent constructs, a larger sample was targeted [63]. Ultimately, 568 valid responses were collected, exceeding the minimum requirements and ensuring adequate statistical power and result robustness.

### Data collection procedure and ethical considerations

This study targeted full-time employees working in the education sector in Guangdong Province, China. Guangdong was selected based on theoretical and practical considerations, as its highly developed economy, competitive labor market, and diverse education system (including public schools, private schools) make employee retention a salient issue.

Respondents were required to (1) be currently employed full-time in the education sector in Guangdong and (2) have at least one year of working experience in their current organization, ensuring sufficient organizational exposure to evaluate retention-related factors.

Data were collected via an online questionnaire administered through the Wenjuanxing platform between September to December 2025. The survey was self-administered, anonymous, and voluntary, and required approximately 10–15 minutes to complete. Two screening questions were used to confirm respondents’ eligibility: (1) Are you currently working in Guangdong Province? (2) Are you currently working in the education sector? Responses with substantial missing values or duplicate entries were removed. After data screening and cleaning, 568 valid questionnaires were retained for analysis.

Ethical approval was obtained from the Ethics Committee of SEGi University, Malaysia (Approval No: SEGiEC/SR/GSB/229/2025–2026). All participants were informed of the study’s purpose, assured of confidentiality and anonymity, and provided digital informed consent prior to participation. The study adhered to the ethical principles of the Declaration of Helsinki (2013). The anonymized dataset and study materials are available as Supporting Information in accordance with PLOS ONE data transparency requirements.

### Data analysis procedures

The data analysis was conducted in two sequential stages.

In Stage 1, data were analyzed using SPSS 27.0 and SmartPLS 4.1. The analysis included assessment of common method bias, evaluation of measurement model reliability and validity, and estimation of the structural model using Partial Least Squares Structural Equation Modeling (PLS-SEM).

In Stage 2, Multi-Group Analysis (MGA) was conducted using SmartPLS 4.1 to examine potential differences in structural relationships across predefined subgroups (e.g., gender or school type). Prior to MGA, measurement invariance was assessed to ensure the comparability of constructs across groups. MGA was then applied to identify whether the effects of key push and pull factors on employee retention varied significantly between groups, thereby uncovering heterogeneity in retention mechanisms. The analysis was conducted using SmartPLS 4.1, and all procedures are reported to ensure transparency and reproducibility.

### Demographic information

Table 1 presents the demographic characteristics of the respondents (N = 568). In terms of age, the majority of participants were between 20 and 39 years old, accounting for 60.2% of the sample, indicating a relatively young and mid-career workforce in the education sector. Respondents aged 40–49 and 50–59 constituted 21.1% and 18.7%, respectively.

**Table 1:** Demographic Information (N = 568).

	Category	Frequency	Percent
Age	20-29	187	32.9
	30-39	155	27.3
	40-49	120	21.1
	50-59	106	18.7
Gender	Male	292	51.4
	Female	276	48.6
School Type	Public School	276	48.6
	Private School	292	51.4
Years of Working Experience	1-2	62	10.9
	2-5	232	40.8
	5-10	202	35.6
	More than 10 years	72	12.7

Regarding gender, the sample was relatively balanced, with 51.4% male and 48.6% female respondents. In terms of school type, 51.4% of respondents worked in private schools, while 48.6% were employed in public schools, suggesting comparable representation across institutional contexts.

With respect to work experience, the majority of respondents had 2–5 years (40.8%) or 5–10 years (35.6%) of experience, indicating substantial professional exposure. Only 10.9% had less than two years of experience, while 12.7% had more than ten years, indicating that the sample included both relatively new entrants and long-tenured employees. Overall, this distribution supports the suitability of the sample for examining employee retention mechanisms in this study.

## Data analysis

### PLS-SEM analysis

#### Common method bias.

The Harman single-factor test was conducted to assess potential common method bias [64]. The results indicated that the largest single factor accounted for 28.65% of the total variance, which is well below the recommended threshold, suggesting that common method bias is unlikely to be a serious concern [65].

Additional, following common practice in PLS-SEM, variance inflation factors (VIF) were also examined. All VIF values were below 3.3 [66], further indicating that common method bias is unlikely to substantially affect the results.

#### Measurement model.

##### Reliability and validity:

The measurement model was evaluated in terms of indicator reliability, internal consistency reliability, and convergent validity [67]. As shown in Table 2, all indicator loadings exceeded the recommended threshold of 0.70, indicating satisfactory indicator reliability [67]. Internal consistency reliability was assessed using Cronbach’s alpha and composite reliability (CR). The Cronbach’s alpha values ranged from 0.793 to 0.884, while CR values ranged from 0.798 to 0.892, all surpassing the recommended minimum level of 0.70, thus demonstrating strong internal consistency reliability across all constructs [67].

**Table 2:** Internal Consistency Reliability and Convergent Validity.

Construct	Items	FL	α	CR	AVE
CO			0.819	0.819	0.648
	CO1	0.833			
	CO2	0.795			
	CO3	0.804			
	CO4	0.788			
EM			0.884	0.887	0.683
	EM1	0.869			
	EM2	0.798			
	EM3	0.805			
	EM4	0.834			
EN			0.793	0.798	0.616
	EN1	0.786			
	EN2	0.779			
	EN3	0.762			
	EN4	0.813			
ER			0.882	0.883	0.739
	ER1	0.863			
	ER2	0.845			
	ER3	0.859			
	ER4	0.871			
JB			0.839	0.892	0.674
	JB1	0.842			
	JB2	0.797			
	JB3	0.817			
	JB4	0.827			
JD			0.869	0.873	0.717
	JD1	0.888			
	JD2	0.845			
	JD3	0.816			
	JD4	0.838			
PI			0.801	0.811	0.714
	PI1	0.866			
	PI2	0.838			
	PI3	0.831			
TD			0.848	0.856	0.687
	TD1	0.869			
	TD2	0.799			
	TD3	0.810			
	TD4	0.835			

Convergent validity was evaluated using the average variance extracted (AVE). The AVE values ranged from 0.616 to 0.739, exceeding the threshold of 0.50, which indicates that each construct explains more than half of the variance of its indicators and confirms adequate convergent validity [67].

Discriminant validity was assessed using both the Fornell-Larcker criterion and the heterotrait-monotrait ratio (HTMT). As shown in Table 3, the square root of the AVE for each construct was greater than its correlations with other constructs, satisfying the Fornell-Larcker criterion [68].

**Table 3:** Fornell-Larcker Criterion.

Construct	CO	EM	EN	ER	JB	JD	PI	TD
CO	0.805							
EM	0.362	0.827						
EN	0.176	0.182	0.785					
ER	0.439	0.456	0.386	0.86				
JB	−0.326	−0.266	−0.14	−0.348	0.821			
JD	−0.386	−0.431	−0.242	−0.567	0.236	0.847		
PI	−0.13	−0.271	−0.109	−0.299	0.099	0.21	0.845	
TD	0.406	0.411	0.251	0.436	−0.327	−0.388	−0.13	0.829

In addition, as shown in Table 4, HTMT values ranged from 0.119 to 0.645, all of which were well below the conservative threshold of 0.85, indicating adequate discriminant validity among all constructs [69]. Together, these results confirm that the constructs in the measurement model are empirically distinct.

**Table 4:** HTMT Value.

Construct	CO	EM	EN	ER	JB	JD	PI	TD
CO								
EM	0.425							
EN	0.219	0.213						
ER	0.516	0.515	0.457					
JB	0.394	0.344	0.172	0.416				
JD	0.456	0.490	0.291	0.645	0.276			
PI	0.155	0.323	0.138	0.352	0.119	0.247		
TD	0.485	0.475	0.301	0.500	0.386	0.451	0.158	

#### Structural model.

Before assessing the structural relationships, collinearity diagnostics were conducted using inner variance inflation factor (VIF) values. The results indicate that all VIF values ranged from 1.077 to 1.449, which are well below the recommended threshold of 5 [66], suggesting that multicollinearity is not a concern in the structural model.

Model fit was assessed using the standardized root mean square residual (SRMR) and the normed fit index (NFI). As shown in Table 5, the SRMR value of 0.042 is well below the recommended threshold of 0.08, indicating a good model fit [70]. In addition, the NFI value of 0.871 exceeds the acceptable cutoff value of 0.80 for PLS-SEM models, suggesting model fit is acceptable [67].

**Table 5:** Model Fit.

	Saturated model
SRMR	0.042
NFI	0.871

#### Results.

**Fig 2:**
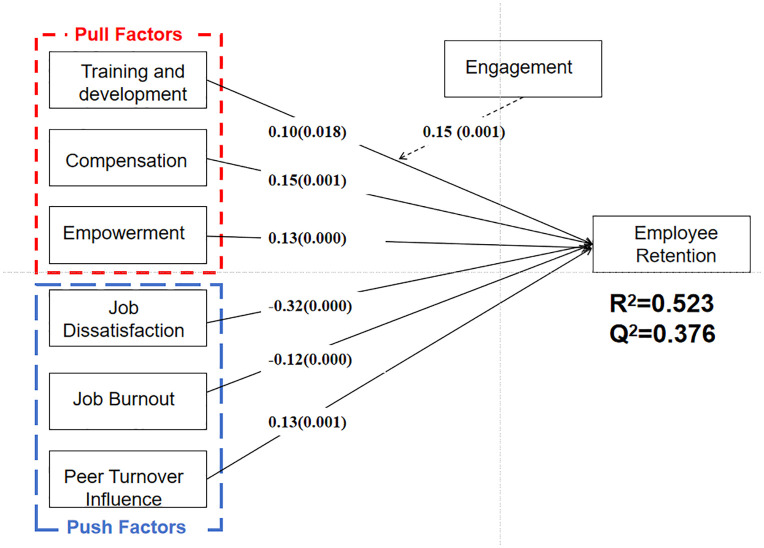
Result of Structure Model.

Fig 2 and Table 6 presented the results of the structural model analysis. The model explained a substantial proportion of variance in employee retention (R^2^ = 0.523) and demonstrated strong predictive relevance (Q^2^ = 0.376). Effect sizes (f^2^) were further assessed to evaluate the contribution of each predictor. Among the predictors, job dissatisfaction (JD) shows the largest effect (f^2^ = 0.147), approaching a medium effect size. Engagement (EN) demonstrates a relatively stronger effect (f^2^ = 0.103) compared to other variables. Other constructs, including compensation (CO; f^2^ = 0.036), empowerment (EM; f^2^ = 0.026), job burnout (JB; f^2^ = 0.027), and peer turnover influence (PI; f^2^ = 0.031), exhibit small effect sizes. Training and development (TD) shows a minimal effect (f^2^ = 0.015) [67].

**Table 6:** Path Coefficient.

	Relationship	β	STDEV	T value	P	Result
H1	TD - > ER	0.10	0.04	2.36	0.018	Supported
H2	CO - > ER	0.15	0.05	3.39	0.001	Supported
H3	EM - > ER	0.13	0.03	3.98	0.000	Supported
H4	JD - > ER	−0.32	0.05	6.84	0.000	Supported
H5	JB - > ER	−0.12	0.03	3.69	0.000	Supported
H6	PI - > ER	−0.13	0.04	3.27	0.001	Supported
H7	EN x TD - > ER	0.15	0.04	3.79	0.000	Supported

Note: * p < 0.05; ** p < 0.01; *** p < 0.001

All hypothesized direct paths were supported. Training and development had a positive effect on employee retention (H1: β = 0.10, p = 0.018). Compensation (H2: β = 0.15, p = 0.001) and empowerment (H3: β = 0.13, p = 0.000) also showed significant positive relationships with employee retention.

Among push factors, job dissatisfaction exhibited the strongest negative effect on employee retention (H4: β = −0.32, p = 0.000), followed by peer turnover influence (H6: β = −0.13, p = 0.001), and job burnout (H5: β = −0.12, p = 0.000).

In addition, the moderation effect between employee retention and training and development was significant (H7: β = 0.15, p = 0.000), indicating that engagement moderates the relationship between training and development and employee retention.

#### Moderation analysis.

**Fig 3:**
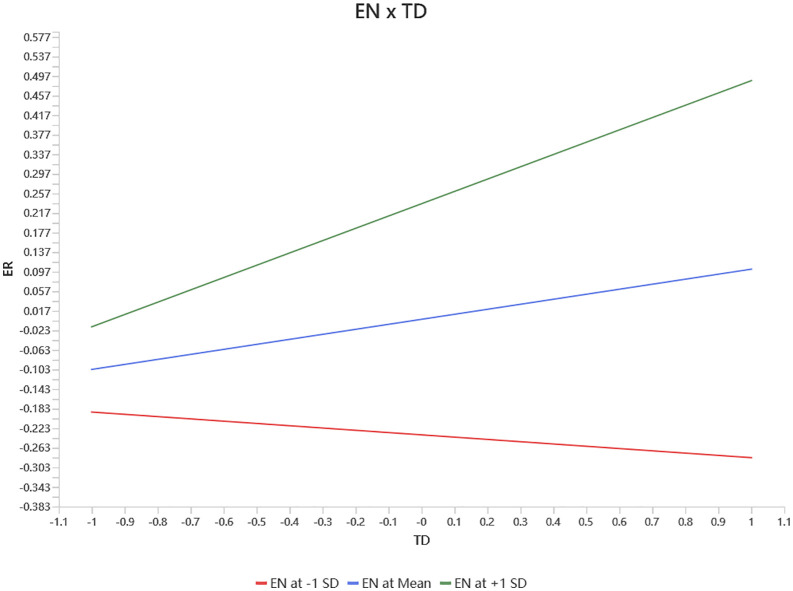
Slope Plots.

The results of the moderation effect tests are summarized in Table 6, with the slope plots presented in Fig 3. As shown in Fig 3, the effect of TD on ER is significantly moderated by EN. When EN is at a high level (+1 SD), TD exhibits a significant and steep positive relationship with ER, indicating that under high EN conditions, an increase in TD markedly enhances ER. In contrast, when EN is at average levels, the positive effect of TD on ER persists but with a markedly reduced slope. Conversely, at low EN levels (−1 SD), a negative relationship emerges between TD and ER, indicating that increased TD actually inhibits ER when EN is low.

### Multi-group analysis

In this study, gender and school type were selected as grouping variables for the multi-group analysis. Gender differences are commonly examined in organizational behavior research because male and female employees may exhibit distinct perceptions of organizational factors and work-related behaviors [71]. Similarly, school type (public vs. private) can reflect differences in institutional culture, resources, and management practices, which may influence employees’ responses [72,73,39].

Before conducting multi-group analysis (MGA), measurement invariance across groups was assessed using the MICOM procedure to ensure the comparability of latent constructs [74]. For both gender groups (male vs. female) and school type groups (public vs. private schools), configural and compositional invariance were established. As shown in Tables 7 and 8, all constructs met the criteria for compositional invariance, with no significant differences observed across groups. Therefore, partial measurement invariance was established, indicating that the measurement models are comparable across groups and enabling valid comparison of structural path coefficients. Consequently, any observed differences in the structural model can be interpreted as reflecting substantive group differences rather than measurement bias.

**Table 7:** Assessment of Measurement Invariance Based on Gender.

Group 1	Con	Conf. Inv.	Comp.Inv.		PMI	Equal Mean		Equal Var		FMI
			C = 1	5% Quantile		Diff.	CI.	Diff.	CI.	
Male vs. Female	CO	Yes	1.000	0.997	Yes	0.092	[−0.161; 0.167]	0.04	[−0.210; 0.197]	Yes
	EM	Yes	0.999	0.998	Yes	0.19	[−0.160; 0.166]	−0.072	[−0.196; 0.191]	Yes
	ER	Yes	1.000	1.000	Yes	0.049	[−0.162; 0.166]	0.077	[−0.204; 0.198]	Yes
	JB	Yes	0.998	0.994	Yes	−0.031	[−0.166; 0.169]	0.147	[−0.193; 0.198]	Yes
	JD	Yes	1.000	0.999	Yes	−0.012	[−0.173; 0.166]	0.033	[−0.197; 0.203]	Yes
	PI	Yes	0.999	0.991	Yes	0.051	[−0.159; 0.161]	−0.152	[−0.273; 0.283]	Yes
	TD	Yes	1.000	0.997	Yes	0.083	[−0.163; 0.161]	0.044	[−0.163; 0.158]	Yes

Note: Con.: Construct; Conf. Inv.: Configural Invariance; Comp. Inv.: Compositional Invariance; CI: Confidence Interval; PMI: Partial Measurement Invariance; FMI: Full Measurement Invariance. “Yes” indicates that invariance criteria are satisfied based on the MICOM procedure.

**Table 8:** Assessment of Measurement Invariance Based on School Type.

Group 2	Con	Conf. Inv.	Comp.Inv.		PMI Est.	Equal Mean		Equal Var		FMI Est.
			C = 1	5% Quantile		Diff.	CI.	Diff.	CI.	
Public School vs. Private School	CO	Yes	0.995	0.994	Yes	−0.189	[−0.196; 0.193]	0.295	[−0.227; 0.270]	Yes
	EM	Yes	0.997	0.996	Yes	0.018	[−0.198; 0.199]	0.109	[−0.215; 0.258]	Yes
	ER	Yes	0.999	0.999	Yes	0.029	[−0.200; 0.201]	0.150	[−0.221; 0.266]	Yes
	JB	Yes	0.997	0.988	Yes	−0.048	[−0.201; 0.204]	0.067	[−0.219; 0.258]	Yes
	JD	Yes	0.997	0.998	Yes	−0.050	[−0.196; 0.202]	0.149	[−0.227; 0.267]	Yes
	PI	Yes	0.997	0.982	Yes	0.201	[−0.201; 0.209]	−0.152	[−0.273; 0.283]	Yes
	TD	Yes	0.996	0.995	Yes	−0.003	[−0.195; 0.208]	0.112	[−0.184; 0.211]	Yes

**Note:** Note: Con.: Construct; Conf. Inv.: Configural Invariance; Comp. Inv.: Compositional Invariance; CI: Confidence Interval; PMI: Partial Measurement Invariance; FMI: Full Measurement Invariance. “Yes” indicates that invariance criteria are satisfied based on the MICOM procedure.

MGA results (in Table 9) indicate that no statistically significant differences were found between male and female groups for any of the structural paths, as all p-values exceeded the 0.05 threshold. Although minor variations in path coefficients were observed across groups, these differences were not statistically meaningful, suggesting that the structural model is invariant across gender.

**Table 9:** Results of Multigroup Analysis.

Relationship	Group 1	β0	β1	Difference	P value	Significant difference
CO - > ER	Male (1) vs Female (0)	0.115*	0.199**	−0.084	0.352	No
EM - > ER		0.149**	0.12*	0.03	0.654	No
JB - > ER		0.247***	0.228***	0.018	0.833	No
JD - > ER		−0.167	−0.081	−0.086	0.185	No
PI - > ER		−0.328***	−0.289***	−0.039	0.679	No
TD - > ER		−0.124**	−0.132**	0.009	0.912	No
Relationship	Group 2	β0	β1	Difference	P value	
CO - > ER	Private School (0) vs Public School (1)	0.136*	0.17**	−0.033	0.714	No
EM - > ER		0.17***	0.094	0.076	0.261	No
JB - > ER		0.313***	0.146**	0.166	0.033	Yes
JD - > ER		−0.108*	−0.131**	0.023	0.728	No
PI - > ER		−0.312***	−0.331***	0.019	0.844	No
TD - > ER		−0.132**	−0.119	−0.013	0.869	No

Note: * p < 0.05; ** p < 0.01; *** p < 0.001

With respect to school type (private vs. public schools), the majority of structural path differences were also not statistically significant. Specifically, compensation, empowerment, job dissatisfaction, peer turnover influence, training and development, and the interaction term did not show significant differences between groups (p > 0.05). However, a statistically significant difference was identified in the relationship between job burnout and employee retention (JB → ER, p < 0.05), indicating that the strength of this effect varies across institutional contexts.

## Discussion

Drawing on the Push-Pull framework, this study employed PLS-SEM and multi-group analysis (MGA) to examine how push and pull factors influence employee retention in the Chinese education sector, using survey data collected from 568 educators in Guangdong Province.

### Influence of pull factors on employee retention

The empirical results indicate that pull factors are positively associated with employee retention in the education sector, reflecting the role of organizational resources in enhancing the perceived value of staying.

#### Training and development.

Training and development are positively associated with employee retention (β = 0.10, p < 0.05), supporting H1. These findings are consistent with prior research showing that career development enhances employees’ perceived benefits of staying [33,31]. From a social exchange perspective, these opportunities demonstrate organizational investment while fostering mutual commitment. From a psychological mechanism perspective, employees interpret training opportunities as long-term career benefits, which increase the perceived opportunity cost of leaving and strengthen retention intentions. Furthermore, MGA results indicate that this relationship remains largely consistent across gender and school type, indicating that training and development function as widely recognized attractiveness factors across educational contexts.

#### Compensation.

Compensation is positively associated with employee retention (β = 0.15, p < 0.01), supporting H2. These results are in line with prior studies indicating that fair and competitive compensation enhances educators’ perceptions of organizational support and reduces turnover intentions [15,10]. MGA results suggest that the effect of compensation on employee retention does not differ significantly across gender groups or school types. Psychologically, compensation reinforces both economic security and fairness perceptions, thereby increasing the attractiveness of remaining in the organization.

#### Empowerment.

Empowerment is positively associated with employee retention (β = 0.13, p < 0.001), supporting H3. This result corroborates prior research suggesting that greater autonomy and decision-making involvement enhance teachers’ professional identity and organizational commitment [42]. From a psychological perspective, empowerment strengthens employees’ sense of control and self-efficacy, which increases emotional attachment to the organization and supports retention decisions. The MGA findings indicate that this relationship remains consistent across diverse groups, highlighting that psychological resources are broadly important in promoting employee retention.

### Influence of push factors on employee retention

The empirical results indicate that push factors are negatively associated with employee retention in the education sector, reflecting how adverse work experiences reduce the perceived desirability of staying.

#### Job dissatisfaction.

Job dissatisfaction is negatively associated with employee retention (β = −0.32, p < 0.001), supporting H4. This finding is consistent with existing literature indicating that dissatisfaction with workload, recognition, and working conditions weakens organizational commitment and accelerates withdrawal intentions [1,49]. The evidence suggests that when employees perceive a mismatch between their expectations and actual job conditions, they are more likely to reconsider their decision to remain in the organization. MGA analysis shows that the effect of job dissatisfaction does not differ significantly across gender and school type, indicating that its influence on employee retention is consistent across different groups.

#### Job burnout.

Job burnout also exhibits a significant negative effect on employee retention (β = −0.12, p < 0.001), supporting H5. This finding is consistent with prior studies indicating that emotional exhaustion and depersonalization may reduce teachers’ psychological resources and long-term occupational sustainability [52,51]. The results suggest that prolonged work strain and emotional exhaustion may reduce employees’ capacity to remain engaged, thereby weakening their intention to stay.

MGA results indicate that this relationship differs significantly across school types, with a stronger effect observed in private schools. This may reflect differences in institutional contexts, such as workload structures and organizational expectations, which may shape how burnout influences retention decisions.

#### Peer turnover influence.

Peer turnover influence demonstrates a significant negative effect on employee retention (β = −0.13, p < 0.01), supporting H6. This result is consistent with social influence and social learning theories, which suggest that colleagues’ departure behaviors shape individual perceptions of organizational stability and career viability (Bandura, 1977; [53]). The evidence indicates that employees may reassess their own decisions when observing others leaving, which can reduce the perceived stability of the organization and increase the likelihood of considering leaving. The MGA analysis shows that the effect of peer influence remains largely consistent across gender and school type, indicating that social comparison processes operate similarly across groups. Within the Push-Pull framework, peer turnover serves as a social push mechanism by normalizing exit behavior and lowering the psychological barriers to leaving, thereby weakening retention intentions..

### Moderating role of engagement

The results indicate that the effect of training and development on employee retention varies across levels of engagement: it is positive at high engagement, weaker at moderate engagement, and becomes negative at low engagement.

This pattern can be explained through a resource interpretation and allocation mechanism. Training and development do not function as inherently beneficial resources; instead, their effects depend on whether employees are willing and able to invest cognitive and emotional resources to utilize them. When engagement is high, employees actively participate in development activities, interpret training as organizational support and career investment, and convert it into personal growth and long-term commitment, thereby strengthening retention [55].

Conversely, when employee engagement is low, their willingness to participate in developmental activities also decreases. In this situation, training may not be viewed as a resource, but rather as an additional source of stress that requires time, energy, and psychological investment. As a result, employees may conserve their energy and exhibit a tendency to withdraw, leading to a negative impact of training on employee retention. This mechanism aligns with the Job Demands-Resources (JD-R) model, which posits that the effectiveness of job resources depends on the employee’s motivational state [11]. Furthermore, it is consistent with the Conservation of Resources (COR) theory, which suggests that when an individual’s resource levels are low, they tend to conserve limited resources and avoid further investment [11]. These findings indicate that employee retention is not only determined by the presence of training and development practices, but also by the underlying psychological mechanisms through which employees interpret and respond to them.

## Significance and limitations

### Theoretical significance

This study extends the Push-Pull framework by simultaneously examining pull factors (training and development, compensation, empowerment) and push factors (job dissatisfaction, burnout, peer turnover influence) in explaining employee retention in the education sector. By integrating insights from the push-pull framework and employee retention literature, this study positions retention as a dynamic outcome shaped by the balance between job resources and job demands. The findings suggest that retention decisions result from the joint effects of attraction and pressure forces rather than isolated factors.

By introducing engagement as a moderating variable, this study further enriches the Push-Pull framework, demonstrating that the effectiveness of organizational resources is contingent upon employees’ psychological engagement. This is consistent with JD-R theory, which emphasizes that job resources are most effective when employees possess sufficient motivational energy to utilize them. In addition, MGA results indicate that only job burnout differs significantly across school types.

These findings highlight that both individual characteristics and institutional conditions shape how employees evaluate organizational resources and work-related pressures, thereby refining the explanatory scope of the Push-Pull framework. Overall, the study contributes to retention literature by bridging motivational and behavioral (Push-Pull-based) explanations of employee retention.

### Practical significance

From a practical perspective, this study offers context-specific implications for school administrators and education policymakers.

First, training and development and compensation are identified as core pull factors, suggesting that schools may prioritize structured career development programs and transparent, equitable compensation systems to support employee retention. Schools are therefore encouraged to implement structured career development pathways, such as regular professional training programs, teaching skill certification systems, and clear promotion criteria. In addition, establishing transparent and performance-linked but fairness-oriented compensation schemes may help strengthen employees’ perceived organizational support and retention intention.

Second, the stronger negative association between burnout and retention in private schools indicates a need for targeted interventions in high-pressure environments, such as workload management policies, mental health support programs, and more balanced performance evaluation systems. Practical measures may include regulating teaching workload distribution, introducing mandatory rest periods, providing access to psychological counseling services, and optimizing performance evaluation systems to reduce excessive performance pressure.

Moreover, the moderating role of engagement highlights that retention-oriented practices are most effective when employees are psychologically engaged in their work. Accordingly, schools may complement structural policies with initiatives that enhance employee involvement, such as participatory decision-making and supportive leadership practices, such as participatory decision-making, regular teacher feedback mechanisms, collaborative teaching culture, and supportive leadership behaviors that strengthen teachers’ sense of belonging and autonomy

Finally, the observed group differences suggest that retention strategies should be adapted to specific employee groups and institutional contexts, rather than adopting a one-size-fits-all approach. For example, private schools may require stronger burnout-prevention policies, whereas public schools may benefit more from enhancing motivation through empowerment and recognition systems, rather than applying uniform retention strategies across institutions.

These implications should be interpreted within the specific context of the education sector and aligned with organizational conditions.

### Limitation

Despite its contributions, this study has several limitations. First, the empirical design is based on a cross-sectional survey, which, by its very nature, does not allow for robust causal inference; future studies may employ longitudinal or experimental designs to better capture dynamic processes and strengthen causal explanations. Second, the reliance on self-reported data may introduce common method bias and response bias. Future research could improve robustness by incorporating objective indicators or multi-source data.

Third, potential endogeneity issues may exist, such as omitted variable bias or reverse causality, which cannot be fully addressed within the current research design. Future studies may apply instrumental variable approaches or longitudinal data to mitigate this concern. Fourth, the sample is confined to the education sector in Guangdong, which may limit the generalizability of the findings. Future studies could extend the model to other sectors or conduct cross-regional comparisons. Finally, additional moderators or mediators—such as organizational culture or leadership style—may be incorporated to further refine and extend the Push-Pull framework.

## Conclusion

The present study provides empirical evidence supporting the Push-Pull framework in explaining employee retention in the education sector. The findings show that pull factors (training and development, compensation, and empowerment) positively influence employee retention, while push factors (job dissatisfaction, burnout, and peer turnover influence) have negative effects. In addition, engagement significantly moderates the relationship between training and development and employee retention.

This study indicates that retention is shaped by the interaction between job resources and job demands rather than by isolated factors. Multi-group analysis further shows that only job burnout differs significantly across school types. Methodologically, the study applies PLS-SEM and MGA with validated measures and a relatively large sample, enhancing the robustness of the findings. From a practical perspective, schools should strengthen career development, compensation fairness, and empowerment, while also address burnout and improve employee engagement to enhance retention outcomes. Overall, this study uses Push-Pull framework to provide a more comprehensive explanation of employee retention and offers practical implications for education management.

## Supporting information

S1 File Checklist Inclusivity in Global Research. (DOCX)

S2 File Dataset. (XLSX)

S3 File Appendix S1.‌‌ (DOCX)
