# Supplementary material for: A study of push and pull factors influencing employee retention in education sector in China: Using PLS-SEM and multi-group analysis
Source: PLoS One. 2026 May 18;21(5):e0349605. doi: 10.1371/journal.pone.0349605 (PMC13183212; doi:10.1371/journal.pone.0349605)
Supplement: S3 File — (DOCX) [file pone.0349605.s003.docx]

**Appendix 1**

| **Abbreviation** | **Full Term** |
| --- | --- |
| PP | Push-Pull |
| ER | Employee Retention |
| TD | Training and Development |
| CO | Compensation |
| EM | Empowerment |
| JD | Job Dissatisfaction |
| JB | Job Burnout |
| PI | Peer Turnover Influence |
| EN | Engagement |
| PLS-SEM | Partial Least Squares Structural Equation Modeling |
| MGA | Multi-Group Analysis |
| β | Path Coefficient |
| SD | Standard Deviation |
